# A Graphene Quantum-Dot Electrometer Array for Sub-Micrometre Imaging of Interfacial Charge Layers in Cryofixed Cells: A Design Analysis

**DOI:** 10.3390/bios16100587

**Published:** 2026-10-19

**Authors:** Cem Celebi, Stefan Ludwig, Afif Siddiki

**Affiliations:** 1 Physics Department, Izmir Institute of Technology, 35430 İzmir, Türkiye; cemcelebi@iyte.edu.tr; 2 Paul-Drude-Institut für Festkörperelektronik, Leibniz-Institut im Forschungsverbund Berlin e.V., Hausvogteiplatz 5-7, 10117 Berlin, Germany; ludwig@pdi-berlin.de; 3 Vocational School, Istanbul Atlas University, Hamidiye, Anadolu Avenue No. 40, 34408 Istanbul, Türkiye

**Keywords:** bilayer graphene, quantum dot, charge sensing, Coulomb blockade, quantum capacitance, surface potential, membrane potential, cryofixation, biosensor

## Abstract

We analyse, as a design study, a cryogenic sensor that images the electrostatic stray field of a vitrified biological cell with an array of gate-defined quantum dots in gapped bilayer graphene. No device has been fabricated and no transport data are reported. The sensor is a Coulomb-blockade electrometer: each dot converts the offset charge induced on it by the sample into a shift in its resonance, and the steepness of that resonance—a property of the discrete density of states rather than of the device area—sets the gain, so that sensitivity is not lost as the element is made smaller. For a 50 nm dot at 4.2 K we estimate a charge sensitivity of order 10^−2^ e/√Hz for a charge 50 nm away, limited by an assumed 1/f offset-charge noise; this is a calculated, not a measured, figure. Because a vitrified sample is electroneutral beyond the Debye length, the sensor does not see net charge but the frozen interfacial dipole layers, whose dipole density equals the permittivity times the potential step across each layer. The measured quantity is therefore a band-limited map of lateral variations in the interfacial potential within about 100 nm of the sensor; a laterally uniform layer is invisible. In this map the membrane potential enters with a weight β = C_m_t/ε_w_ε_0_ ≈ 0.07 relative to the surface potential of the glycocalyx, so the sensor is primarily a surface-potential imager and only secondarily, through a calibration we specify, a membrane-potential probe. A 10 mV surface-potential contrast at a period of 250 nm exceeds the 1 ms detection limit by a factor of about 4–13, and the 8 × 8 array at 250 nm pitch resolves periods of 250–300 nm. We give the device geometry, the plunge-freezing protocol, the controls and the inversion procedure, and list what remains to be measured.

## 1. Introduction

For many years, it was thought that quantum mechanical effects could not be observed in biological systems because of rapid decoherence at room temperature and the great complexity of living systems [1]. In contrast, more recent studies [2] have triggered a controversial discussion about quantum mechanical phenomena observed at cryogenic temperatures [3,4] and at room temperature [5] (CT and RT, respectively). A point often overlooked in this debate is the possibility of significantly improving detection sensitivity for biological systems, particularly at the nanoscale, by using energy quantisation and coherent dynamics in the sensor’s quantum regime.

The explanation that follows details how the sensitivity arising from energy quantisation can be applied to biological material; in particular, it describes a non-contact method for investigating the charge distribution in a single cell. We distinguish carefully between charge and potential. In the medical literature the quantity of interest is a potential—the membrane potential or the action potential—whereas an electrostatic sensor couples to charge. The two are connected, but not in the simple way in which a net charge produces a potential: a cell in electrolyte, and equally a vitrified cell, is electroneutral on scales larger than the Debye length, and what reaches a sensor outside it is the field of its interfacial dipole layers. Section 4.2 makes this connection quantitative and states which potentials the sensor measures, and with what weight.

The paper is structured as follows: we first review the current capabilities in cellular electrophysiology and identify the limits of resolution of the available techniques, before explaining quantised charge transport in graphene and the basis of the sensing mechanism. We then provide a quantitative assessment of the sensitivity and resolution that can be achieved, and outline the device geometry and fabrication process, the cryopreservation procedure, the measurement method, and the self-consistent electrostatic calculation needed to invert the measured signal. To keep the presentation unambiguous we state at the outset that the paper analyses a single device—an array of Coulomb-blockade quantum dots in gapped bilayer graphene—and that the monolayer quantum-Hall point contact is discussed only as an alternative (Section 3 and Section 5.4); every number in the paper refers to the dot array. Throughout the text, we make a distinction between parts that are in agreement with established physics and those that still need to be verified experimentally.

## 2. The Latest Development in the Measurement of the Electromagnetic Properties of Cells

**Figure 1:**
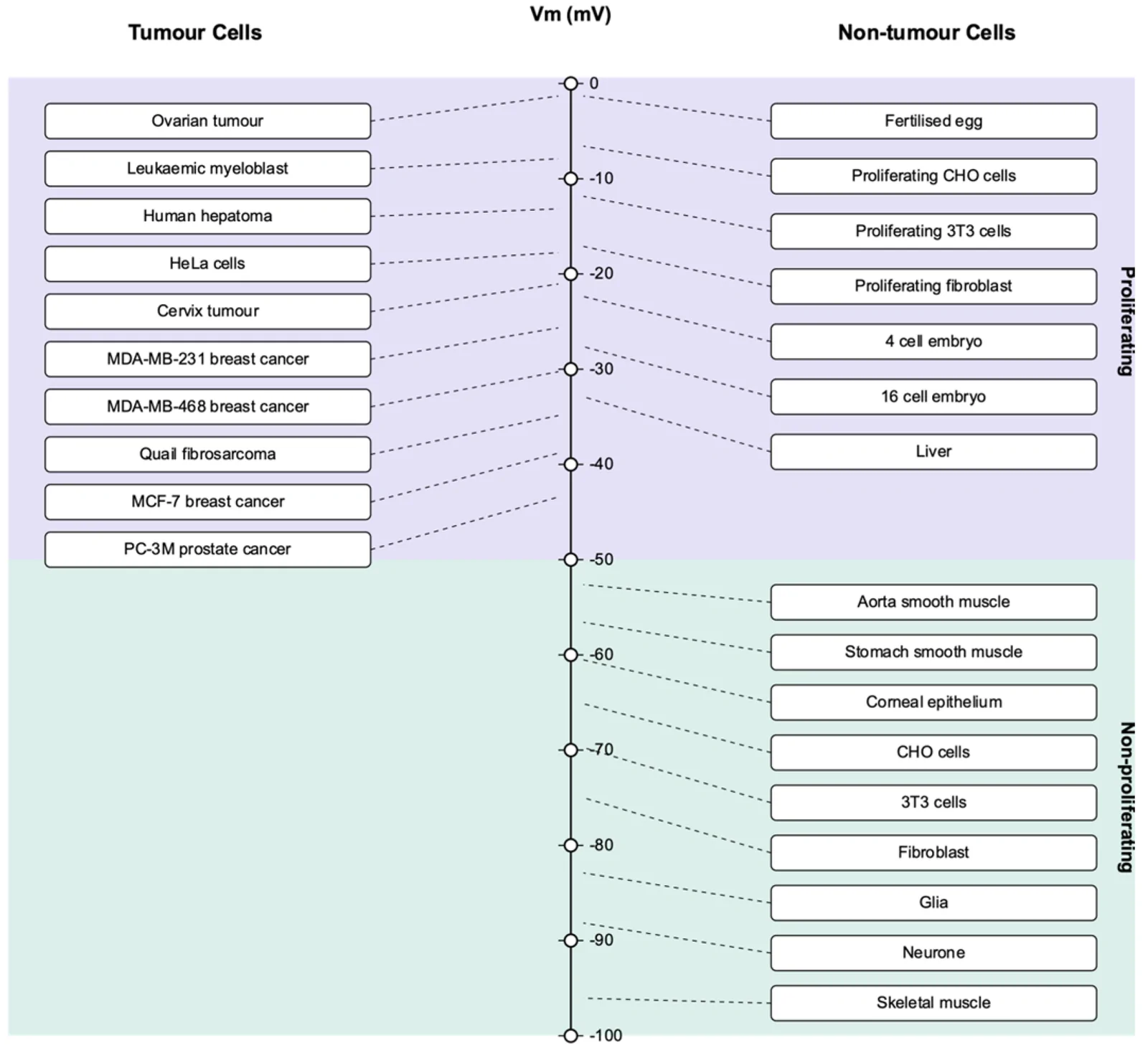
The (resting) membrane potentials of tumour and non-tumour cells along with their features regarding proliferation. This figure is reproduced from Ref. [7] under the terms of the Creative Commons Attribution licence. These values motivate the measurement; as shown in Section 4.2, the proposed sensor accesses the membrane potential only indirectly, with a weight β ≈ 0.07 relative to the surface potential.

Cancer, fertilisation and dementia are all among the major health problems of our time and are expected to remain so as the World Health Organisation [6] has stated. These conditions are generally thought to be closely associated with intra- and intercellular charge transport. By examining the bioelectric properties of cells, we can better understand various aspects of cell physiology, including the formation of electric fields across cell membranes and charge transport through them. At the most basic level, the membrane potential is the potential difference between the inside and the outside of the cell. Understanding the resting membrane potential (RMP) is essential to comprehend the regulation of circadian rhythms and the development of both cancer and stem cell therapies [7]; Figure 1 summarises RMP values for different cell types.

**Figure 2:**
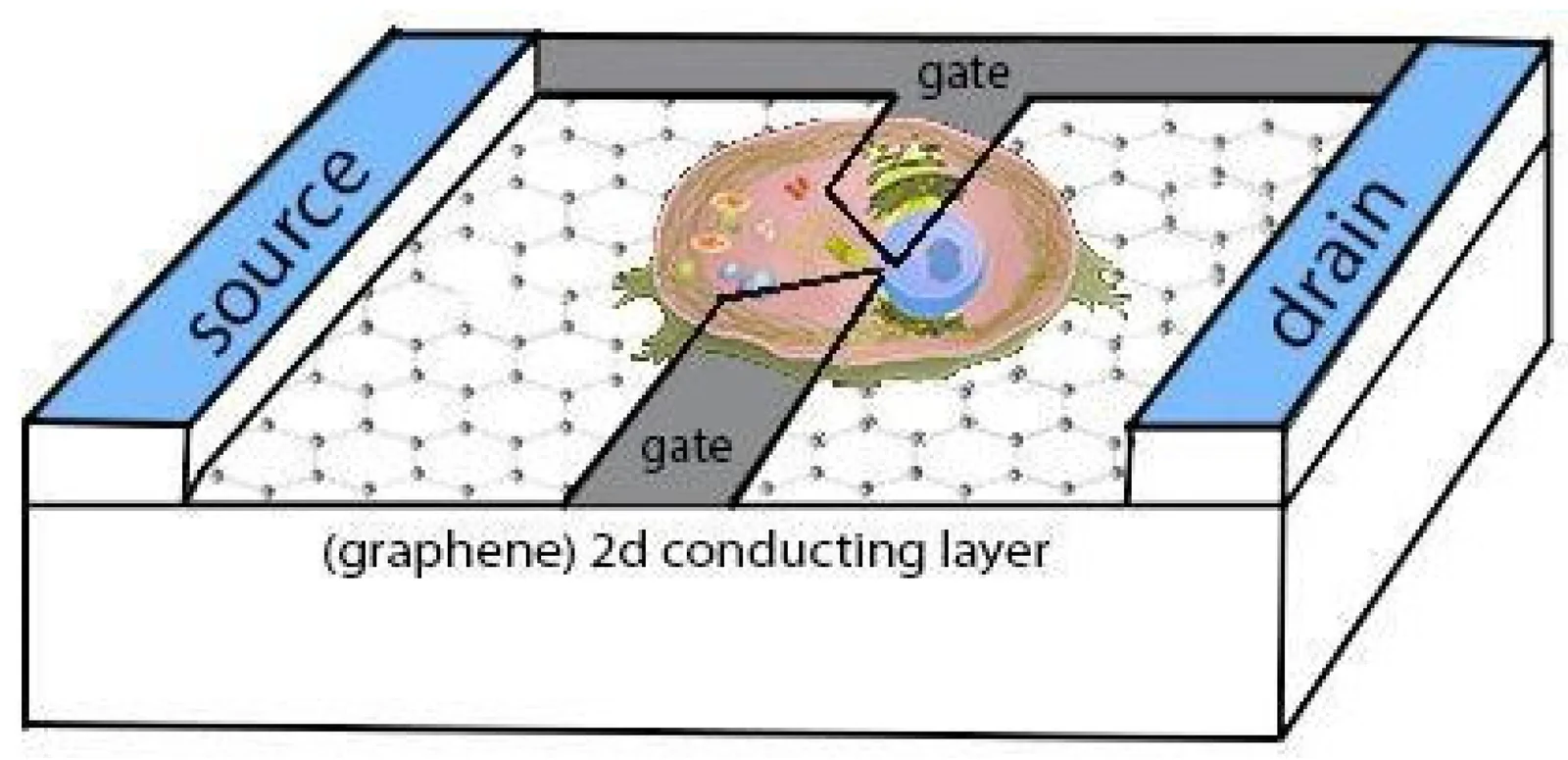
A sketch showing a cell placed directly onto a graphene-based quantum sensor (not to scale); the sensor is a quantum point contact with its constriction defined by lithography and adjusted using metal split gates (grey) which are situated beside, not above, the sensing area.

To study cellular electrophysiology, various methods have been developed to examine the nanoscale electromagnetic characteristics of biological systems. The most well-known of these is the patch-clamp technique [8,9,10], for which the Nobel Prize in Physiology or Medicine was awarded in 1991 [11]. The resolution of this technique is limited by pipette size and by technical factors such as damage to the electrical seal, and the pipette can damage the cell. Other approaches—dielectrophoresis, impedance spectroscopy and electroporation—provide better resolution and sensitivity [12,13,14], but their sensitivity drops rapidly when the probe is made smaller than the micrometre scale. Methods using a local probe, such as atomic force and scanning probe microscopy, improve this and have been used to make impedance and capacitance measurements on isolated systems such as bilayer lipid membranes [15]; nevertheless, these are also invasive and affected by geometry-dependent size effects. A second group of techniques completely eliminates the need for a mechanical probe. Graphene solution-gated field-effect transistor arrays can record extracellular action potentials from electrogenic cells at room temperature with a high signal-to-noise ratio [16,17,18], and nitrogen-vacancy centres in diamond can detect the magnetic field of single-neuron action potentials under ambient conditions [19]. Since both of these methods are used on living cells, they are complementary rather than competitive: they measure the time-dependent transmembrane potential or the current associated with it, averaged over a device or sensing volume of micrometre scale, while the sensor proposed here focuses on the static charge distribution with a resolution determined by tens of nanometres. The closest analogy in terms of resolution and charge sensitivity is the scanning single-electron transistor, which can map static fields and charges with a resolution of about 100 nm and a sensitivity of the order of 0.01e [20], but this requires a scanned cryogenic probe to be placed above the sample. A sensor whose sensitivity does not decrease as the probe is made smaller and which does not require scanning would therefore have a unique place among these techniques; this is the approach taken in the present case (Figure 2).

## 3. Quantised Charge Transport: An Overview of the Background and Current State of the Art

Two-dimensional (2D) conducting layers are ideal for the investigation of quantum effects [21]. Graphene, which consists of a single layer of carbon atoms arranged in a hexagonal lattice, is especially suitable to be used as the base material in a quantum sensor since it features weak scattering of charge carriers together with a Landau-level spacing large enough for quantum Hall effects to persist at higher temperatures [22,23], and because it is chemically inert and biocompatible, allowing cells to be grown or placed directly onto the enclosing layer [16,17,24,25,26]. Quantum dots (QDs) and quantum point contacts (QPCs) are created by confining the charge carriers laterally—through etching, by means of the electric field effect, or using a magnetic field—into regions that are comparable to the de Broglie wavelength of the carriers, thereby quantising both their energies and their transport. Figure 2 depicts a QPC coupled to a cell.

The conductance of a quantum point contact is determined by the width of the constriction and can be adjusted by the voltages applied to the metallic gates [27]. In the quantum Hall regime, a second and independent quantisation takes place when a strong perpendicular magnetic field is applied to the conducting layer [28,29]. In graphene this quantisation is due to the Landau levels of massless Dirac fermions, the spacing between the N = 0 and N = 1 levels being

(1) ΔE = vF (2eℏB)^1/2^

where e is the elementary charge, ℏ the reduced Planck constant and B the magnetic flux density; the Fermi velocity vF ≈ 1 × 10^6^ m/s is obtained from the band structure of graphene, and, except for a small logarithmic renormalisation at low density, it does not depend on the carrier density, so that when gating is applied the Fermi energy is shifted and as a result it is the particular Landau level that lies at E_F_ which is selected rather than the Fermi velocity being changed. Since ΔE increases with the square root of B and reaches about 115 meV at a field of 10 T—this being roughly seven times the cyclotron energy ℏω_c_ ≈ 17 meV of a GaAs two-dimensional electron system at the same field—quantised transport in graphene continues to occur at considerably higher temperatures than in conventional semiconductor heterostructures, and the quantum Hall effect has been observed in graphene at room temperature, although only at magnetic fields of the order of 30 T [23].

A feature of monolayer graphene determines the shape of the device and it should be mentioned before the sensor is described. Since monolayer graphene has no band gap, a negatively biased split gate does not deplete the sheet but instead forms a p–n junction, and carriers arriving normally are transmitted through this junction with certainty—this is known as Klein tunnelling [30]. However, etched constrictions eliminate this effect but do so at the cost of introducing edge disorder, which leads to localised states and transport resembling Coulomb blockade rather than clear conductance quantisation [31]. Two conclusions can be drawn. In the quantum Hall regime the confinement is due to the magnetic field and not to the gates, transport takes place in edge channels, and the split gate regulates the transmission of these channels; it follows that QPC operation in monolayer graphene is well defined at the operating point under consideration here. In the case of the quantum-dot variant, which depends on Coulomb blockade rather than on Landau quantisation, a band gap is necessary, and bilayer graphene with a gap induced by a displacement field is the suitable platform, on which gate-defined quantum point contacts and dots with clean quantisation have been demonstrated [32,33,34,35,36,37,38]. The drawback is that the dispersion is parabolic and as a result the spacing between Landau levels is smaller, which weakens the argument at high temperatures that was originally a reason for choosing graphene. We therefore suggest using monolayer graphene for the quantum-Hall QPC and bilayer graphene for the Coulomb-blockade dot.

The plan is to employ one cell as a local top gate for a nanoscale quantum sensor and to measure the change in current that occurs through the constriction as a result. If there is an array of constrictions situated below the cell, this gives a map of the stray field across the sensor area; as illustrated in Section 4.1, the exponential decrease in that field with depth means that the map is limited to the charge located within about 100 nm of the graphene sheet.

## 4. Sensing Mechanism: From a Steep Density of States to a Steep Electrometer Response

The most advanced techniques currently used for investigating the electrophysiological properties of biomaterials usually involve measuring the impedance across membranes or the capacitance between a cell and a fine tip. Although these methods provide high spatial resolution, this resolution is generally achieved by destroying the cell. The sensor discussed in this case instead seeks to combine non-contact detection of the interfacial charge layers of a cell with sub-micrometre resolution.

Operationally, the quantity read out from each element is not a capacitance. Each dot is held at a working point on the flank of a Coulomb resonance by a feedback loop acting on its plunger gate; the cell shifts the resonance, and the recorded signal of element j is the gate-voltage correction ΔV_j_ or, equivalently, the offset charge Q_0,j_ = C_g_ΔV_j_ induced on the island, with C_g_ the plunger-gate capacitance. The transconductance ∂I/∂Q_0_ is the gain of this conversion and, together with the noise, fixes the sensitivity, but it is not itself the signal. Because the frozen sample is static, the measurement is a static offset measurement: offsets larger than one Coulomb period are obtained by counting resonances, and the background offsets of the bare elements must be known (Section 5.3). The quantum capacitance enters through the density of states, which sets how steep the resonance is, as follows.

The capacitance between the sensor and the biomaterial, which itself depends on the charge distribution in the cell, determines the current that is detected by the sensor. In the case of a planar capacitor consisting of two parallel metal plates each of area A, with a separation distance d and in a medium having a permittivity ε = ε_0_ε_r_, the geometric capacitance is given by C_G_ ≃ εA/d, on the condition that both plates have a large and continuous density of states (DOS). However, when one of the plates is replaced by a material that has a quantised and discrete density of states D(E), this description no longer applies and the quantum capacitance C_Q_ = e^2^D(E) has to be taken into account [39,40]. The function D(E) varies with the dimensionality as well as with the applied electric and magnetic fields. In the QPC and QD detectors being considered here, lateral confinement causes a discrete density of states to appear in the graphene sheet; alternatively, a perpendicular magnetic field arranges the density of states in the graphene into Landau levels.

(2) EN = sgn(N) (2eℏvF^2^|N|B)^1/2^, N = 0, ±1, ±2, …

Positive N labels electrons and negative N holes, N = 0 being the graphene-specific zero-energy level, and vF is the Fermi velocity introduced above; Equation (1) gives the energy spacing between the N = 0 and N = 1 rungs of this ladder. If the Fermi energy lies between two neighbouring Landau levels the density of states vanishes, D(E) → 0, and thus in a clean sample at low temperature the quantum capacitance also tends to zero; in practice finite temperature and disorder broaden the levels and D(E) remains small but non-zero [41,42]. Throughout, D(E) denotes the total density of states of the confined region, in states per unit energy, so that CQ = e^2^D(E) is a capacitance; where an extended sheet is meant we write DA(E) for the density of states per unit area and CQA = e^2^DA(E) for the corresponding capacitance per unit area. Equation (7) below follows from the former with D ≈ 1/δE. The capacitance detected by the sensor is the result of the series combination [39].

(3) C_tot_ = C_G_ C_Q_/(C_G_ + C_Q_).

It is important to be exact about where the advantage of the quantum regime consists, since the simple argument does not hold. The geometric capacitance is proportional to the area of the constriction, and so reducing the size of a classical capacitive probe decreases the signal it can produce; one might then hope that a large quantum capacitance would make up for this. However, Equation (3) does not allow this: for capacitances in series, C_tot_ ≤ min(C_G_, C_Q_), which means that no value of C_Q_ can raise the total capacitance above its geometric upper limit, and thus the sensor cannot exceed the performance of a classical detector of the same area in terms of absolute capacitance. The measurement, on the other hand, is not sensitive to Ctot but to the change in Ctot when the cell’s stray field is present, and it is here that the quantised density of states plays a decisive role. As E_F_ is swept across a Landau level, D(E) increases from a minimum limited by disorder and temperature to a sharp maximum over an energy scale determined by the level width Γ rather than by ΔE. Near such a resonance, dC_tot_/dE_F_ is large, so a small shift in the local electrostatic potential caused by the cell results in a considerable change in the transmission of the constriction and therefore in the measured current. Two further points are relevant to scaling. First, the coupling of the cell to the sensor occurs through the lever arm α = C_cell_/C_Σ_, that is, the ratio of the cell-to-device capacitance to the total device capacitance; since all the capacitances in the problem scale linearly with the device dimension, α is approximately scale-invariant, and the potential shift per elementary charge in the cell therefore does not decrease as the device becomes smaller. Second, the figure of merit is the transconductance dI/dV at the operating point, and the fundamental limit is not transconductance but charge sensitivity: since a smaller device carries a smaller current, the cost of miniaturisation is paid in terms of integration time rather than in signal amplitude. Section 4.1 gives both statements a quantitative form. The same sharpness is observed in real space as in energy: in the quantum Hall regime a gated two-dimensional electron system breaks down into compressible and incompressible regions, and across the boundaries between these regions the local density of states—and with it the local quantum capacitance—changes abruptly on a nanometre scale [43,44]. It is precisely by operating the constriction at such a boundary that a weak external field is turned into a large signal.

The same principles of electrostatics apply to the spatial resolution. A charge distribution at a depth z below the surface of the cell gives rise at the sensor to a stray field whose Fourier component with in-plane wavevector q is reduced by a factor of exp(−qz)/ε(q). Features smaller than the separation are thus suppressed exponentially and the resolution that can be achieved is determined by the distance between the sensor and the charge rather than by the sensor alone. In the case where the cell is placed directly on the encapsulated graphene sheet this distance is of the order of 10 to 100 nm. The resolution that follows is not of the order of the separation itself: it is set jointly by the roll-off of the forward kernel of the split-gate geometry and by the pitch at which the array samples the plane, and is of order 250 nm, as quantified in Section 5.5; this is also the reason for constructing the device in graphene rather than in a buried semiconductor heterostructure, in which the two-dimensional electron system is located much deeper below the surface. This same exponential factor is also the main limitation of the method and it must be mentioned from the beginning. For the wavevectors which contain information about spatial scales of sub-micrometre size, q ≳ 2π/(300 nm), the factor exp(−qz) reduces the contribution of a charge at z = 1 µm by some eight orders of magnitude compared to that of a charge at z = 50 nm. Consequently, a single planar sensor array is unable to reconstruct a three-dimensional charge map of a cell having a thickness of a micrometre. All it can measure is a two-dimensional map of the charge within about 100 nm of the graphene sheet, with a stronger weighting towards the surface; this is the observable that we claim below.

### 4.1. Sensitivity and Resolution: A Quantitative Estimate

All numerical estimates in this section refer to the Coulomb-blockade quantum dot defined in gapped bilayer graphene. The monolayer quantum-Hall point contact operates by a different mechanism—transmission through a magnetically confined constriction rather than resonant tunnelling through a charged island—and has a different capacitance network, level width and operating field; we do not attach a sensitivity figure to it here, and the comparison with scanning single-electron-transistor electrometry made below applies to the dot alone.

In order to clarify the matter, we calculate the response of a quantum-dot sensor to a single elementary charge located in the cell. We consider an island with a lateral dimension L ≈ 50 nm, which is defined in encapsulated graphene, having a total island capacitance CΣ ≈ 10 aF, and a separation z ≈ 50 nm between the island and the charge, in a medium with a relative permittivity εr ≈ 3—this value is suitable for both h-BN and ice at 4.2 K. The charging energy is then given by EC = e^2^/CΣ ≈ 16 meV, which is considerably greater than kBT ≈ 0.36 meV at 4.2 K, indicating that Coulomb blockade is well established. The capacitance between the island and a patch of cell of the same lateral size is Ccell ≈ ε_0_εrL^2^/z ≈ 1.3 aF, so that the lever arm α = Ccell/CΣ ≈ 0.13.

The coupling of an external charge to the island is described by a single dimensionless quantity, the weighting potential φw(r), defined as the potential at r when the island is held at unit potential with every other conductor grounded. An elementary charge at r then produces an offset charge q_0_ = eφw on the island and shifts its addition energy by δE = ECφw, with EC = e^2^/CΣ. This replaces the two-step estimate used previously, in which the bare Coulomb potential of the point charge was evaluated at the island and then multiplied by a lever arm; the two factors are not independent, and using both counts part of the coupling twice. Two independent estimates of φw agree at the design point: treating the cell patch as an electrode of area L^2^ at distance z gives φw ≈ Ccell/(4πε_0_εrz) = 0.080, and the split-gate aperture of width d = 100 nm beneath h ≈ 25 nm of h-BN transmits with weight exp(−πh/d) ≈ 0.46, which after geometric decay over z = 50 nm is of the same order. We adopt φw = 0.08 for the estimates that follow. A numerical solution of Laplace’s equation for the actual split-gate geometry, reported in Section 5.5, gives a larger value, 0.39 at this design point in a treatment that takes the constriction to be uniform along its length; since a three-dimensional element of finite length couples more weakly than an infinitely long slit, we retain the smaller value here and note that the sensitivity figures below are correspondingly conservative, by up to a factor of five. With φw = 0.08, δE = 1.3 meV. At 4.2 K the thermal linewidth is Γ = 3.5 kBT = 1.3 meV, the full width at half maximum of a thermally broadened single-level Coulomb-blockade resonance [45,46,47], so a single elementary charge at 50 nm shifts the resonance by approximately one linewidth; at 300 mK in a helium-3 system the same charge corresponds to more than ten linewidths.

The corresponding charge sensitivity follows from the slope of the Coulomb peak. A linewidth Γ corresponds to a width ΔQ = eΓ/EC = 0.08 e in offset charge, so with a peak current Ipeak = 100 pA the maximum transconductance is |∂I/∂q_0_| ≈ 0.4 Ipeak/ΔQ ≈ 5 × 10^2^ pA per elementary charge on the island. Against a transimpedance-amplifier noise floor of 100 fA/√Hz this gives 2 × 10^−4^ e/√Hz referred to the island, or 2.5 × 10^−3^ e/√Hz referred to an external charge at 50 nm. This white-noise value is a ceiling rather than a working figure: gate-defined quantum dots exhibit 1/f offset-charge noise which dominates below a few kilohertz, and assuming a level of order 10^−3^ e/√Hz at 1 Hz—representative of dots in semiconductor heterostructures, and to our knowledge not yet established for encapsulated bilayer graphene—the external-charge sensitivity in the band of interest degrades to order 10^−2^ e/√Hz. It is this conservative figure that we compare with the 0.01e sensitivity reported for the scanning single-electron transistor [20], at a smaller sensor-to-charge distance and without a scanned probe; establishing the 1/f level in graphene is among the first measurements the proposed device would have to deliver.

For a boxcar average of duration τ the equivalent noise bandwidth is 1/(2τ), so the minimum detectable external charge is qmin = Sq/√(2τ). With Sq = 10^−2^ e/√Hz this is 0.22 e at τ = 1 ms: a single elementary charge at 50 nm is detected with a signal-to-noise ratio of about 4.5 in 1 ms, reaches unit signal-to-noise in approximately 50 µs, and reaches a ratio of ten in approximately 5 ms. Averaging beyond roughly 0.1 s brings no further improvement once the 1/f contribution dominates.

Three noise sources must be distinguished, and their hierarchy determines what limits the measurement. Shot noise on the sensor current is the smallest: for Ipeak = 100 pA the full Poissonian value √(2eI) is 5.7 fA/√Hz, and the Fano factor of a tunnel-coupled dot, or of graphene near the Dirac point, reduces it further [48], so it lies almost a factor of twenty below the 100 fA/√Hz floor of a conventional transimpedance amplifier. The amplifier therefore sets the white-noise ceiling quoted above; were it improved to the shot-noise limit the external-charge sensitivity would reach 1.4 × 10^−4^ e/√Hz. Neither is the binding constraint in practice, because graphene devices are dominated at low frequency by flicker noise: 1/f fluctuations in graphene and in graphene-on-h-BN structures are well documented and depend strongly on the dielectric environment and on interface quality [49]. This is why the estimate above is referred to an assumed 1/f offset-charge level rather than to the amplifier, and why the graphite back gate of Section 5.2 is preferred to an oxide-supported one. Measuring the 1/f level of the encapsulated bilayer stack is therefore the first characterisation the device requires, and it is the quantity that would revise the sensitivity figure in either direction.

Two comments help to place this figure in context. To begin with, the signal is not that of an isolated point charge. A vitrified cell is electroneutral beyond the Debye length, so the relevant source is the frozen dipole layer analysed in Section 4.2; for a 10 mV contrast in surface potential at a period of 250 nm it is equivalent to about three elementary charges per 50 nm × 50 nm footprint, a factor of about 4–13 above the minimum detectable charge of 0.22 e in 1 ms. The measurement is therefore close enough to the sensitivity limit that millivolt contrasts require averaging, and the remaining limitations are systematic: the background of the vitrified buffer, the static background offsets of the elements, the screening through the split-gate aperture discussed in Section 5.2, and the ill-posed nature of the inversion. Secondly, the estimate is based on the assumption of an unscreened point charge in a uniform dielectric; the graphene sheet itself screens the field, the frozen cell is dielectrically inhomogeneous, and the split gates function as a partial ground plane; each of these factors reduces the coupling, and a self-consistent calculation of the type described in Section 5.1 must be carried out before the figure can be considered to be better than by an order of magnitude. We make this point explicitly because it is the order of magnitude—rather than just the mechanism—that decides whether or not the experiment is worth carrying out.

### 4.2. What the Sensor Measures: Interfacial Dipole Layers, Surface Potential and Membrane Potential

The estimate of Section 4.1 refers to a point charge, but a frozen cell does not present the sensor with isolated charges. In physiological electrolyte every fixed charge—the sialic-acid groups of the glycocalyx, the charged head groups of the membrane and the charges that sustain the membrane potential—is neutralised by mobile counter-ions within a Debye length, λ_D_ ≈ 0.8 nm, and plunge freezing arrests these ions in place. On the length scale of the sensor, z_0_ ≈ 50 nm ≫ λ_D_, the sample is therefore electroneutral, and its leading electrostatic moment is the dipole density p(r) of its interfacial layers, directed normal to the sensor plane. For a laterally uniform layer of permittivity ε, Poisson’s equation fixes this dipole density exactly: p = εΔΨ, where ΔΨ is the potential step across the layer. The dipole density is set by the positions of the charges and is preserved on freezing; the potential step is not, since the permittivity falls from that of water to that of the frozen medium. The relevant quantity is thus the potential step in the living state multiplied by the permittivity in which it was established.

Two layers dominate within roughly 100 nm of the sensor. The first is the diffuse layer and glycocalyx at the outer cell surface, for which p_s_ = ε_w_ε_0_ψ_s_, with ε_w_ ≈ 80 and ψ_s_ the surface (Donnan) potential, of order −10 to −30 mV for mammalian cells and close to the electrophoretic zeta potential of about −15 mV for the human erythrocyte. The second is the membrane itself, across which the charge C_m_V_m_ is separated by the membrane thickness t ≈ 5 nm, so that p_m_ = C_m_tV_m_, with C_m_ ≈ 1 µF cm^−2^. Hence,

(4) p(r) = ε_w_ε_0_ [ψ_s_(r) + βV_m_(r)], β = C_m_t/(ε_w_ε_0_) ≈ 0.07.

The membrane potential therefore enters the measured quantity with about one fourteenth of the weight of the surface potential: the erythrocyte resting potential of about −10 mV contributes as much as a 0.7 mV change in surface potential, and a hyperpolarisation to −70 mV as much as 5 mV. By reciprocity, the offset charge induced on element j by a dipole sheet at height z_0_ is

(5) Q_0,j_ = ∫d^2^r p(r) ∂_z_φ_w_^(j)^(r, z_0_),

with φ_w_^(j)^ the weighting potential of Section 4.1 (the sign depends on the orientation chosen for p). Above the electrodes φ_w_ is harmonic, so in Fourier space ∂_z_ → −q and a dipole sheet is equivalent to a charge sheet of density σ_eff_(q) = q p(q). Two consequences follow. First, a laterally uniform dipole layer (q = 0) produces no signal at all: the uniform part of ψ_s_ and of V_m_, which is most of it, is invisible, and the sensor registers only their lateral variations—charge patches, membrane domains and the edge of the cell. Second, a modulation of amplitude δψ at period λ is equivalent to the charge density:

(6) σ_eff_ = (2π/λ) ε_w_ε_0_ δψ.

For δψ = 10 mV and λ = 250 nm, σ_eff_ = 1.8 × 10^−4^ C m^−2^, or about 2.8 e per 50 nm × 50 nm footprint. This, and not the bare fixed charge of the glycocalyx, is the signal to be compared with the sensitivity of Section 4.1. It exceeds the minimum detectable external charge of 0.22 e in 1 ms by a factor of about 13, or of about 4 if the kernel attenuation exp(−qz_0_) ≈ 0.3 at this period is applied in addition to the point-charge coupling φ_w_; the two bracket the true value until the three-dimensional kernel of Section 5.5 is available. A contrast of 1 mV therefore requires averaging over 10–100 ms, close to the point at which 1/f noise stops further gain. The contribution of the membrane potential is smaller by β: a change in V_m_ by 50 mV, uniform over the cell, is invisible beneath the cell and appears only at its edge, where it is equivalent to a step of 3.5 mV in surface potential.

Equation (4) is the relationship between the measured quantity and the membrane potential, and it makes three testable statements. (i) The sensor is primarily an imager of lateral variations in the surface potential at the cell–substrate interface, not a membrane-potential probe. (ii) The membrane potential is accessible only through its contribution at the edge of the cell, with weight β ≈ 0.07, and only by comparison between cells, since a single frozen cell provides a single value of V_m_; the calibration of Section 5.3 is designed accordingly. (iii) During a plunge lasting some milliseconds the ions can still move over distances much larger than λ_D_, so the frozen dipole density is that of the double layers at the temperature at which ionic mobility is arrested, not necessarily that at 300 K; applying Equation (4) with physiological values is therefore an assumption, and it is precisely the assumption that the calibration tests. This narrows the scope relative to the motivation of Section 2: the resting-membrane-potential data of Figure 1 motivate the measurement, but they are not what it delivers.

Lower panel: transmission of a single graphene quantum point contact at B = 2 T. Left: energy dependence of the transmission T(E) for zero gate voltage and for a gate-induced barrier of Vg = 150 meV; the gate raises the subband thresholds and reduces the number of transmitted modes. Right: dependence of the transmission on gate voltage at fixed energies E = 105 meV and E = 61 meV; increasing the gate voltage progressively pinches off the constriction, and the weak non-monotonic feature at high energy is due to mode mixing and resonant scattering in the finite constriction.

## 5. Methodology

We now give the numerical framework required for interpreting a measurement, describe the method to be followed in fabricating the devices, outline the procedure for preparing the biological samples, and explain the transport measurement itself. At this stage no devices have in fact been fabricated and no transport data are provided; the sections on fabrication and measurement refer to a protocol that is proposed and which is based on procedures already in use in our laboratories for graphene devices.

### 5.1. The Numerical Simulation of Graphene-Based Quantum Sensors Coupled with Biomaterials

In order to turn a measured current into a charge distribution, the coupled quantum and electrostatic problem has to be solved self-consistently. This is difficult for two reasons: the electrostatic interaction has a long range and decreases only algebraically with distance, and the response is strongly non-linear due to the fact that the quantised density of states has a sharp, comb-like form. A method which was developed for exactly this type of problem has been used in the case of semiconductor heterostructures and converges much better than conventional methods [50]. In order to apply it to graphene it is necessary to replace the parabolic-band density of states with the Dirac expression—linear in |E| at zero field and composed of the Landau levels of Equation (2) in a perpendicular magnetic field—and then re-derive the resulting non-linear Poisson problem in the same way; the self-consistent problem must be solved before the measurement can be inverted. The two software packages used are KWANT (Version 1.5.0), which is used for quantum transport in open systems [51], and PESCADO (Version 3.0), the electrostatic solver of Ref. [50], which deals with both the Poisson and the generalised Helmholtz equation. Discretising the Dirac Hamiltonian requires care, since a naive finite-difference scheme on a square lattice produces spurious low-energy replicas—the fermion-doubling problem. We therefore follow one of the established remedies: either a real-space scheme constructed to avoid doubling [52], or a reciprocal-space formulation in which the transverse modes are treated in momentum space [53], the latter also accommodating the perpendicular magnetic field required here.

### 5.2. The Fabrication of Graphene Quantum Sensors

The graphene layers are produced by mechanical exfoliation and are then placed on a degenerately doped Si wafer which has a 285 nm thick thermal SiO_2_ oxide; the oxide thickness is chosen for the optical contrast it gives, and the substrate serves for identification and handling rather than as the operating gate. The single-layer areas are initially detected using optical microscopy before being confirmed by single-point Raman spectroscopy and micro-Raman mapping to ensure that they have a monolayer thickness and are uniform.

**Figure 3:**
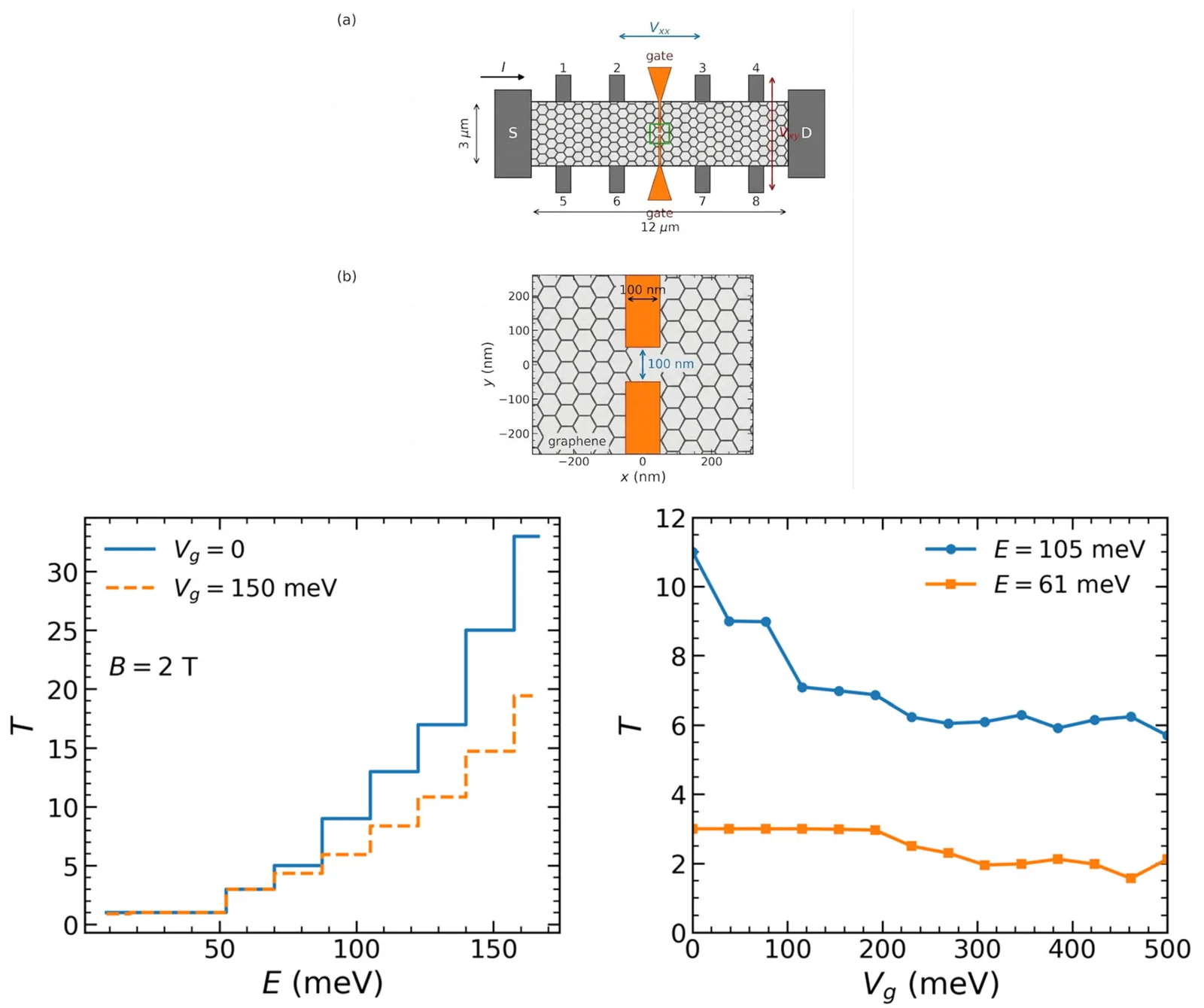
Schematic of the QPC, with the graphene areas shaded in light grey in both panel (**a**) and panel (**b**), the single-layer graphene being represented by a dark grey two-dimensional hexagonal honeycomb pattern, along with the gates (orange) and the contacts (dark grey); the source and drain contacts are connected to the graphene/h-BN heterostructure, and two split gates define one of the apertures with a width d; the frozen cell is positioned directly above this ungated aperture so that its stray field can interact with the QPC without being screened by a metal.

The device is fabricated using electron-beam lithography (EBL). The Cr/Au source and drain electrodes are deposited at the edges of the graphene, which is enclosed in a multilayer hexagonal boron nitride (h-BN) layer [54,55] grown by chemical-vapour deposition. Split-gate electrodes, separated by a nanoscale gap, are then patterned onto the graphene/h-BN stack by EBL. A graphite flake beneath the lower h-BN layer serves as the back gate. This is preferred to the degenerately doped silicon substrate: the graphite gate lies within tens of nanometres of the sheet rather than 285 nm away, giving a far stronger lever arm at modest voltages, and it screens the charge traps at the SiO_2_ interface that are a principal source of 1/f noise. Since the charge sensitivity estimated in Section 4.1 is limited by 1/f offset-charge noise rather than by the amplifier, this is a design choice with a direct effect on the figure of merit; the doped substrate is retained only as an optional global trim electrode. Figure 3 displays the resulting split-gate configuration. The position of the gates is not merely a minor feature but rather a design constraint: any metal situated between the cell and the graphene shields the stray field, so it is necessary for the cell to be placed above the gap between the split gates rather than over the metal. A grounded plane located at a height h above the plane transmits the field through a slit of width d with an attenuation of the order of exp(−πh/d) for the lowest Fourier component that is transmitted, so that with an h-BN thickness h approximately equal to 25 nm and a gate gap d approximately equal to 100 nm about half of the field reaches the graphene, while when d is less than or equal to h the coupling is suppressed exponentially. The gap width, the h-BN thickness and the constriction width therefore have to be optimised together. All electrical contacts are tested at room temperature on a probe station before cooling down. The preparation of the graphene, the fabrication of the device and the room-temperature characterisation follow the procedures that have been established previously [24,56].

We specify the first-generation array explicitly. Sixty-four gapped-bilayer dots are arranged on an 8 × 8 grid of pitch 250 nm, giving an active area of 2 µm × 2 µm—a sub-cellular patch rather than a whole cell. Each aperture is d = 100 nm wide beneath h = 25 nm of h-BN. Source lines are shared along rows and drain lines along columns, so that the array requires 8 + 8 transport lines in addition to gate and back-gate lines, well within the 48–96 line capacity of a standard helium-3 cryostat loom; elements are addressed one at a time. With each element read in feedback for 0.1–1 s, a complete 64-element map takes about one to two minutes. Dissipation is negligible: 64 elements at 100 pA and 200 µV bias amount to 1.3 pW, against the hundred microwatts of cooling power available at 300 mK, so the thermal budget is set by the wiring heat load rather than by the sensors. Gate crosstalk is likewise small, since the potential of a gate decays laterally in the graphene plane over a scale set by h; with a pitch of 250 nm and h = 25 nm the coupling to a neighbouring constriction is negligible, and the back gate acts globally, setting the operating density rather than a local perturbation. Covering an entire cell at this pitch would require of order 10^3^ elements and therefore on-chip cryogenic multiplexing, which we do not address here.

Two material questions bear directly on whether the resonances are sharp enough for the estimates of Section 4.1. Multilayer h-BN grown by chemical-vapour deposition is convenient but is not equivalent to exfoliated crystals in dielectric quality, and transfer residues at the graphene interface are a known source of charge noise; the 1/f level assumed in Section 4.1 is the quantity most sensitive to both, and we regard its characterisation in this stack as a prerequisite measurement rather than an implementation detail. The gate metal bounding each aperture also perturbs the ideal-slit electrostatics assumed in the analytic kernel of Section 5.5, and is included in the numerical weighting potential rather than treated as an ideal slit. Alignment and retention are handled before freezing: lithographic fiducials on the chip allow a cell to be positioned optically over the array to within an optical wavelength, optical tweezers give finer placement, and after freezing the registration is recovered from the array response itself, since the measured signal envelope locates the cell relative to the elements.

### 5.3. Getting the Biomaterials Ready for Measurement at Cryogenic Temperatures

The problem of freezing cells and tissues without damaging them has lasted for over seventy years [57,58,59]. In order to avoid chemical changes that would affect cell properties, it is necessary to control the water content so that lethal ice formation does not occur either inside or outside the cell; a cryoprotecting agent (CPA) is conventionally employed to control ice formation, water transport and nucleation [60]. We take the human erythrocyte as the model cell. It is anucleate and free of internal organelles, it is thin enough to vitrify without a cryoprotectant, it adheres flat to a planar substrate, and its surface charge is dominated by the sialic acid of the glycocalyx and is independently characterised by electrophoretic mobility, so that the total charge in the sampled patch is known in advance to within a factor of order unity.

Washed erythrocytes in isotonic phosphate-buffered saline are deposited on the chip at a dilution giving fewer than one cell per 100 µm^2^, positioned over the array as described in Section 5.2, blotted to a residual film below 1 µm, and plunge-frozen in liquid ethane at 90 K. For films of this thickness the cooling rate exceeds 10^4^ K s^−1^, which vitrifies the sample without a cryoprotectant and therefore without the ionic background that a concentrated agent would introduce. This replaces the slow-cooling protocol assumed in earlier versions of this design: slow cooling at about 1 K per minute dehydrates the cell osmotically and produces extracellular ice with solute exclusion, so that the frozen charge distribution it leaves is that of a dehydrated cell in equilibrium with a concentrated residual solution, which is not the state we wish to image [61]. The sample is transferred under liquid nitrogen and held below 130 K throughout to avoid devitrification before being cooled to the operating temperature. There is no warming step; the sample is not recovered. Isolation of the cells is verified optically before freezing. Clustering does not corrupt the signal—a cell lying above another is beyond the exp(−qz_0_) range and contributes nothing—but it removes the well-defined interface separation on which the reconstruction depends and makes the attribution of a map to a single cell ambiguous; since the array samples an area much smaller than one cell, there is no advantage that would offset this.

**Figure 4:**
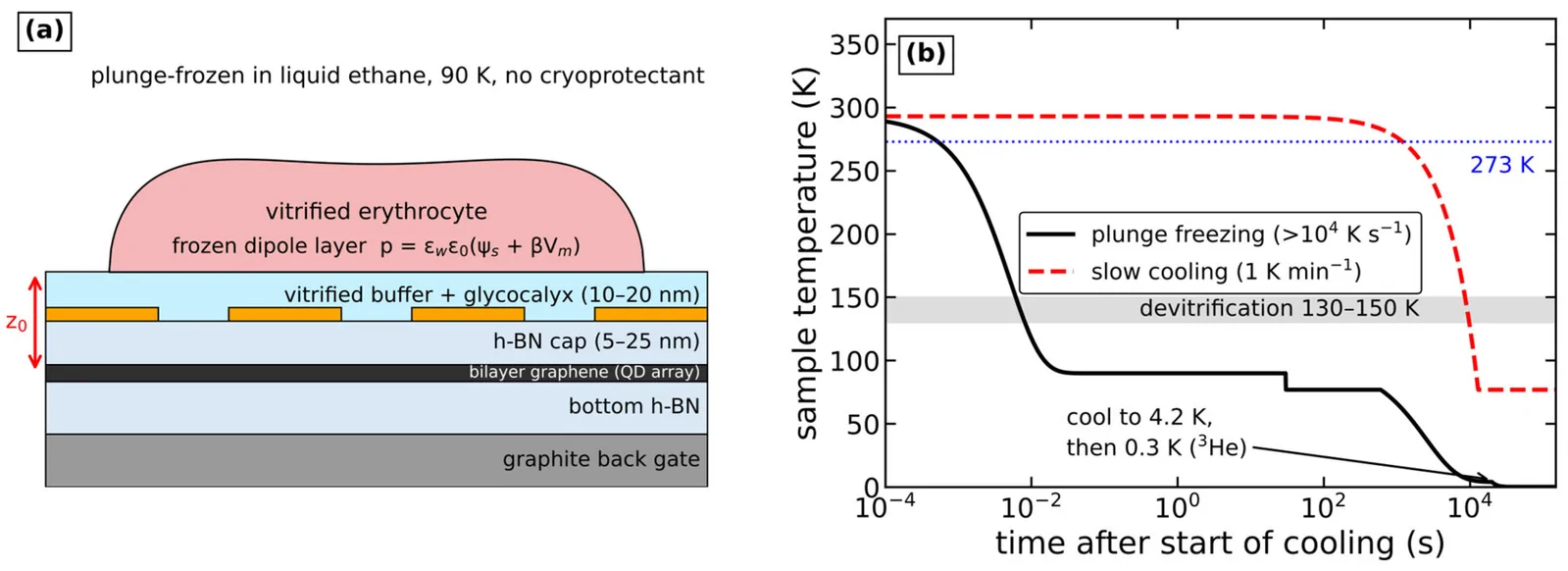
(**a**) Schematic cross-section (not to scale) of a vitrified erythrocyte on the sensor array: graphite back gate, bottom h-BN, gapped bilayer graphene containing the quantum-dot array, h-BN cap (5–25 nm) carrying the split gates (orange), and the residual vitrified buffer and glycocalyx (10–20 nm) beneath the cell. The sensor-to-charge separation z_0_ ≈ 30–50 nm is the sum of the last two layers, and the cell enters the measurement through its frozen interfacial dipole layer, Equation (4). (**b**) Thermal history of the sample for plunge freezing in liquid ethane (black; initial cooling rate above 10^4^ K s^−1^, transfer under liquid nitrogen, cool-down to 4.2 K and then to 0.3 K in the cryostat), compared with slow cooling at 1 K min^−1^ (red dashed), the protocol of earlier versions of this design. The grey band marks the devitrification range 130–150 K and the dotted line 273 K. The durations of the transfer and of the cryostat cool-down are illustrative.

Figure 4 summarises the sample geometry and its thermal history. Panel (a) shows the layer sequence between the quantum-dot array and the cell, from which the separation z_0_ discussed below follows; panel (b) contrasts the plunge-freezing protocol with the slow-cooling protocol it replaces. With plunge freezing the sample passes from 273 K to below the devitrification range within a few milliseconds, too fast for ice to nucleate in a film below 1 µm, and it is kept below 130 K from then on; with slow cooling it spends tens of minutes in the range in which extracellular ice grows and the cell dehydrates.

The device is not restricted to this cell. Because the observable is the lateral variation in the interfacial potential within roughly 100 nm of the sensor plane (Section 4.2), the signal is set by surface-potential contrasts and not by the size of the cell; cell size enters only through the array area required for coverage. Reported zeta potentials of the human erythrocyte (7–8 µm in diameter and about 2 µm thick), of adherent mammalian cells such as HEK293 or fibroblasts (15–20 µm) and of bacteria such as Escherichia coli (1 × 2 µm) lie typically between −10 and −50 mV; the contrasts that the array can image are fractions of these, and a 10 mV contrast at a period of 250 nm is detected with a signal-to-noise ratio of about 4–13 in 1 ms (Section 4.2). Oocytes (100–120 µm) are similar per unit area but far too large for the first-generation array. The margin is about one order of magnitude, not several, so the measurement is limited jointly by sensitivity for millivolt contrasts and by systematic effects—the vitrified-buffer background, the background offsets of the elements, the aperture screening and the conditioning of the inversion.

The expected sensor-to-charge separation is the sum of the h-BN cap, 5–25 nm, and the residual water and glycocalyx layer, 10–20 nm, giving z_0_ ≈ 30–50 nm. This is not assumed but measured: the knee in the measured spatial-frequency spectrum is set by exp(−qz_0_), so the array determines its own z_0_ from the roll-off of the reconstructed map.

Two mechanical problems occur during cool-down and have not yet been investigated experimentally. The thermal contraction of the frozen cell and of the surrounding ice is different from that of the h-BN/graphene stack, which means that strain or delamination at the interface is possible and would alter the effective separation z_0_ that determines both the resolution and the signal; this is measurable rather than merely arguable, and we propose to bound it by thermally cycling a reference charged monolayer of known density. And once the sample is cold the cell can no longer be moved: optical tweezers only function before freezing, so the lateral sampling of the stray field must be carried out by means of an array of constrictions on a single sheet (Figure 3) rather than by repositioning the cell between measurements.

It must be made clear what a measurement on a frozen cell can and cannot establish. The resting membrane potential shown in Figure 1 is a dynamic steady state which is maintained by active ion pumps; it does not persist when the cell is cooled and therefore a cell at 4.2 K has no membrane potential in the physiological sense. What is retained—provided that lethal ice formation does not occur—is the spatial distribution of the charged species, that is to say the ionic gradients and the charged groups of the membrane proteins and nucleic acids, all of which are frozen in place at the moment of solidification. The quantity measured by this sensor is thus a static charge distribution characteristic of the cell state at the instant of freezing, not the electrophysiology of a living cell. Three consequences arise. The first is that the vitrified buffer itself contributes a background—its residual salt and any solute redistribution during the plunge—which must be determined separately by measuring a vitrified buffer film without a cell. Second, the dielectric response of the frozen medium is not that of water: the orientational relaxation of ice is frozen out well above 4.2 K, so only the high-frequency response with ε_r_ ≈ 3, similar to that of h-BN, remains, and, once ionic mobility is quenched, the frozen gap between cell and sensor no longer screens the field of the cell’s interfacial dipole layers, which in liquid electrolyte would be suppressed within a Debye length. Freezing does not, however, turn the cell into a set of bare charges: the counter-ions are frozen in their screening positions, which is why the observable is the dipole density of Section 4.2. Freezing is therefore not just a compromise to allow the sensor to operate at a low temperature; it is in fact what makes an electrostatic measurement possible at all. Third, any comparison with the membrane-potential data given in Ref. [7] is indirect and is based on the assumption that the frozen charge distribution reflects the electrochemical state of the cell before freezing; Equation (4) makes the comparison quantitative, the membrane potential entering with weight β ≈ 0.07 and only where the cell edge lies over the array. Testing that assumption—for example by freezing genetically identical cells that are held at different and independently measured membrane potentials—must be carried out before any biological interpretation can be made and is not discussed here. For the same reason, we call the measurement non-contact rather than non-invasive: no probe penetrates the cell, but the preparation is still destructive.

The background of the vitrified buffer needs a quantitative criterion for acceptance rather than a qualitative warning, since it can reach the sensor more effectively than the signal of interest. A laterally uniform layer contributes only at q = 0 and is invisible to the array, being absorbed into the operating point. What is not invisible is lateral structure from residual salt, from any partial crystallisation and from solute redistribution during the plunge, whose scale is typically of the order of a micrometre; because of the attenuation factor exp(−qz) this long-wavelength structure reaches the graphene essentially unaltered, while 100 nm features of the cell do not. The discriminant is therefore spectral rather than amplitude-based: a vitrified buffer film is measured alone, on the same array and with the same protocol, and its spatial power spectrum is reported together with that of the cell; the measurement is interpreted only in the band where the cell spectrum exceeds the buffer spectrum, which we expect to be q ≳ 2π/500 nm. Where the two spectra overlap in the band that carries the cell signal, it is the freezing protocol that must be changed, not the regularisation.

Four controls are required before any map is interpreted biologically. First, the chip with a vitrified buffer film and no cell, to establish the background. Second, two successive cool-downs of a bare chip, to measure the cycle-to-cycle reproducibility δQ_bg_ of the background offsets of the elements; since a 1 mV contrast corresponds to about 0.3 e (Section 4.2), millivolt-level maps require δQ_bg_ below this value, and elements that the cell does not cover serve in every subsequent run as an in situ reference for drift. Third, a thermal cycle on a reference charged monolayer of known density, to bound interfacial strain and delamination. Fourth, and decisive for the biological claim, a calibration of Equation (4): genetically identical erythrocytes are treated with valinomycin in isotonic media in which extracellular K^+^ is exchanged for Na^+^ at constant ionic strength, so that V_m_ is set close to the potassium equilibrium potential, between about 0 and −90 mV, and measured independently before freezing, while ψ_s_ is held fixed. The same series without valinomycin, in which V_m_ of the erythrocyte changes little, separates a genuine V_m_ dependence from any direct effect of the medium on ψ_s_. Equation (4) predicts that the potential step at the cell edge shifts by about 3.5 mV per 50 mV change in V_m_; with a cell-to-cell scatter of the edge step of 1.5–3 mV, of order ten cells per condition suffice to detect this at three standard errors. If the edge step does not follow V_m_ as predicted, the premise that the frozen state retains the membrane potential fails, and this must be established before any correlation with the disease associations of Ref. [7] is attempted.

**Figure 5:**
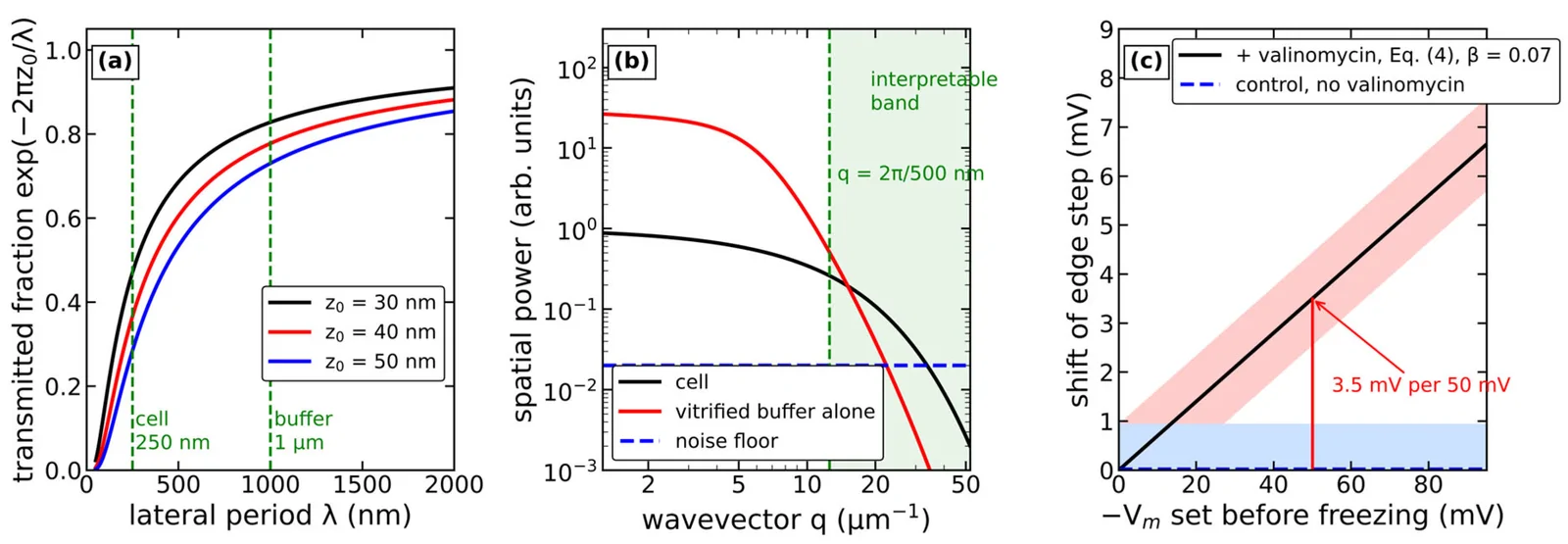
(**a**) Fraction exp(−2πz_0_/λ) of a lateral modulation of period λ that reaches the sensor plane, for z_0_ = 30, 40 and 50 nm. Micrometre-scale structure in the vitrified buffer (1 µm) is transmitted almost unattenuated (0.73–0.83), whereas cell features at 250 nm are reduced to 0.28–0.47. (**b**) The spectral acceptance criterion, illustrated with model spectra: spatial power spectrum of the cell map (black) and of a vitrified buffer film measured alone on the same array (red), both including the kernel attenuation for z_0_ = 50 nm, together with a white noise floor (blue dashed). The map is interpreted only where the cell spectrum exceeds both the buffer spectrum and the noise, which we expect for q ≳ 2π/500 nm (shaded). The spectral shapes are illustrative and not computed for a specific sample. (**c**) Predicted outcome of the calibration of Equation (4): shift in the potential step at the cell edge as a function of the membrane potential V_m_ set with valinomycin before freezing (black; slope β ≈ 0.07, i.e., 3.5 mV per 50 mV), and for the control series without valinomycin (blue dashed). The shaded bands show ±1 standard error for ten cells per condition and a cell-to-cell scatter of 3 mV, the upper end of the range of 1.5–3 mV.

Figure 5 illustrates the quantitative elements of this section. Panel (a) shows why the background of the vitrified buffer has to be separated spectrally rather than by amplitude: at z_0_ = 50 nm a modulation of period 1 µm reaches the sensor plane with 73% of its amplitude, and one of period 250 nm with only 28%. Panel (b) shows the acceptance criterion applied to model spectra of a cell and of a buffer film measured alone, and panel (c) shows the outcome of the calibration of Equation (4) that the valinomycin series and its control are expected to give.

### 5.4. Measurements of Quantum Transport at Low Temperature and High Magnetic Field

A quantum point contact (QPC) acts as a one-dimensional (1D) constriction in a two-dimensional system and exhibits quantised conductance as a result of the lateral confinement. In the case of graphene, the type of charge carrier can be adjusted so that the current is carried either by electrons or by holes. This applies to the gapped bilayer constriction, and to the monolayer constriction only in the quantum Hall regime, since a gapless monolayer constriction at zero field is not pinched off (Section 3). When the QPC is below the first conductance quantum, it becomes a one-dimensional electrostatic barrier, the height of which is directly influenced by the stray field of the neighbouring cell, the cell thus functioning as a variable gate. At cryogenic temperatures the transport through the barrier occurs by quantum tunnelling and the probability of tunnelling increases exponentially as the height of the barrier decreases. At higher temperatures, carriers which have been thermally excited pass over the barrier and the current then becomes less dependent on the height of the barrier.

The lower panel of Figure 3 shows the transmission of a single constriction of this kind. It is computed on the honeycomb tight-binding lattice, with the magnetic field introduced through the Peierls substitution, the constriction defined by the potential of the split gates, and the transmission obtained from the scattering matrix; because the calculation is carried out on the physical lattice rather than on a discretised continuum Dirac equation, the fermion-doubling problem discussed in Section 5.1 does not arise here. The left-hand plot gives the energy dependence of the transmission with and without a gate-induced barrier, and the right-hand plot its dependence on gate voltage at fixed energy, showing the progressive pinch-off on which the point-contact variant relies.

A quantum dot (QD) is made up of a small conducting island which is connected to the leads via quantum point contacts that have been adjusted into the tunnelling regime. Due to the lateral confinement, the carriers have a discrete, atom-like energy spectrum and the positions of the energy levels are shifted by the stray field of the neighbouring cell. At cryogenic temperatures, Coulomb blockade causes current to flow only when a state of the dot is resonant with the chemical potential of the leads, thereby allowing individual carriers to tunnel onto and off the island one at a time. Since the energy levels of the dot are greatly affected by the stray field, the quantum dot functions as a highly sensitive electrometer [62,63,64,65,66]. The lateral confinement determines the quantum capacitance of the dot via the single-particle level spacing δE, a value that is determined by the size of the island and which should not be confused with the Landau gap ΔE of Equation (1). When averaged over a number of levels the density of states is approximately D ≈ 1/δE, so that

(7) C_Q_ ≈ e^2^/δE.

This average value is incorporated into the sensitivity estimate in Section 4.1. At resonance the density of states is greater than this average, rising to a maximum of D ≈ 1/(πΓ), the level width Γ being determined by both the tunnel coupling to the leads and by the temperature. The ratio δE/Γ gives an indication of how quickly C_Q_ changes as the dot levels are shifted by the cell’s stray field, and thus determines the transconductance available at the operating point. Making the island smaller increases δE and reduces the average quantum capacitance, but it also sharpens the resonances; since these effects work in opposite directions, the optimum represents a design compromise rather than a fundamental limit.

**Figure 6:**
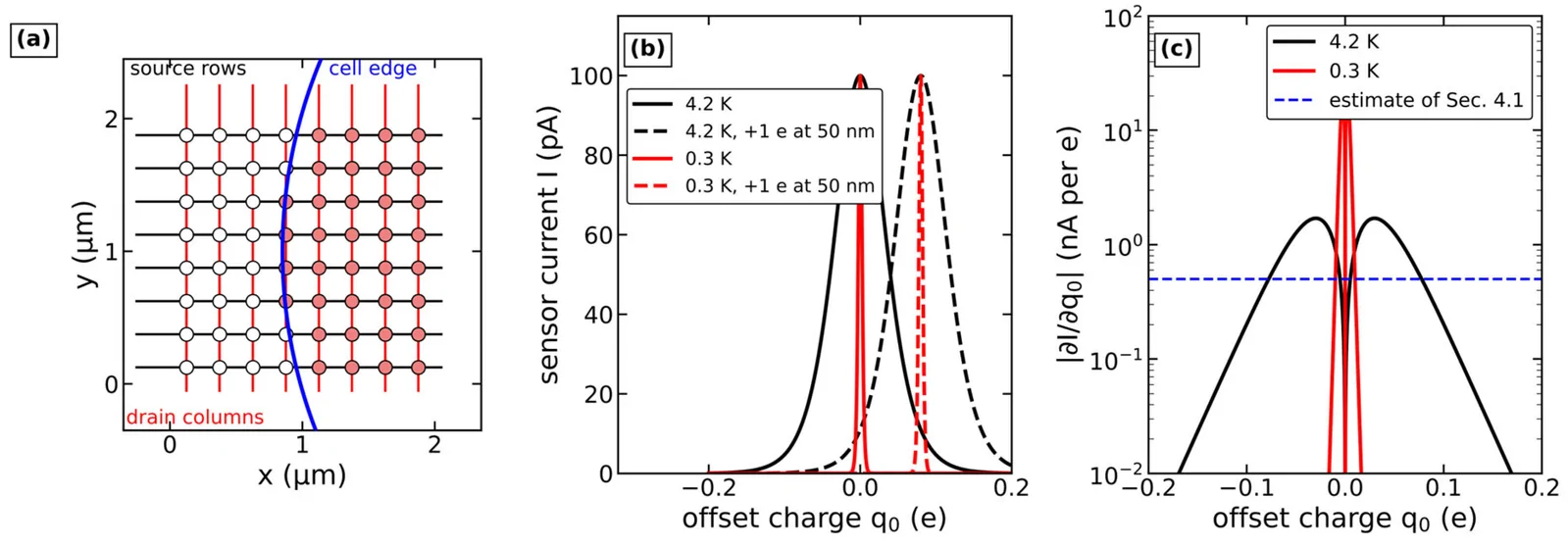
(**a**) Layout of the first-generation 8 × 8 array (pitch 250 nm, active area 2 µm × 2 µm). Source lines are shared along rows (black) and drain lines along columns (red), and the elements are addressed one at a time. Elements beneath the cell (filled) and outside it (open) are separated by the cell edge (blue), where the membrane potential contributes with weight β (Section 4.2); the uncovered elements serve as in situ drift references. (**b**) Thermally broadened Coulomb-blockade resonance of one element, I = I_peak_ cosh^−2^(E_C_q_0_/2ek_B_T), with I_peak_ = 100 pA and E_C_ = 16 meV, at 4.2 K (black) and 0.3 K (red), without (solid) and with (dashed) an elementary charge at 50 nm, which shifts the resonance by φ_w_e = 0.08e. (**c**) Transconductance |∂I/∂q_0_| of the resonances in (**b**). Its maximum on the flank, where the feedback loop holds the working point, is 1.7 nA per e at 4.2 K and 24 nA per e at 0.3 K; the estimate of Section 4.1, 0.5 nA per e (blue dashed), is conservative by a factor of about three.

Figure 6 shows how the array is read out and why the dot is the more sensitive element. Elements are addressed one at a time through the shared row and column lines (Figure 6a), and each is held in feedback at the steepest point of the flank of its Coulomb resonance (Figure 6b). An elementary charge 50 nm above an element shifts the resonance by φ_w_e = 0.08e, which is about one linewidth at 4.2 K and about fourteen linewidths at 0.3 K. For the thermally broadened line shape the maximum transconductance (Figure 6c) is 1.36 I_peak_/ΔQ, i.e., 1.7 nA per elementary charge at 4.2 K; the value 0.4 I_peak_/ΔQ used in Section 4.1 is therefore conservative by a factor of about three, and we retain it.

A QD has a steeper resonance and is thus more sensitive than a QPC, while the QPC is the simpler device and remains functional at higher temperatures. Both types of device can be fabricated on the same sheet and used as sensors. The sensitivity can be increased even more by substituting a single QD with two tunnel-coupled QDs, the coherent charge dynamics of which produce a sharper response; nevertheless, we point out that in order to operate a double dot as a coherent charge qubit it is necessary to have charging energies and coherence times that have not yet been demonstrated in graphene at 4.2 K, so this version should be considered a longer-term possibility rather than something included in the current design.

The cryogenic measurements are made in a helium-bath cryostat fitted with a helium-3 evaporation refrigerator, employing standard quantum transport equipment—namely low-noise voltage sources, lock-in amplifiers, sensitive current amplifiers and a radio-frequency source—together with a superconducting magnet [67,68].

The operating window follows from two independent requirements. The sensor requires kBT ≪ EC: with CΣ = 10 aF the charging energy is 16.0 meV, giving EC/kBT = 44 at 4.2 K and an upper bound near 19 K for the present island size, which a smaller island would raise—an island of 5 nm would place the ceiling near 190 K. Sensitivity improves monotonically as the temperature falls, since the resonance width is Γ = 3.5 kBT, and the helium-3 stage at 300 mK gains an order of magnitude over 4.2 K. The sample imposes the second and more fundamental requirement. In a physiological electrolyte at 300 K the Debye length is 0.79 nm at an ionic strength of 150 mM, so the stray field of a charge 50 nm from the sensor is screened by some twenty-eight orders of magnitude; the measurement is possible only once ionic mobility is quenched, and freezing is therefore not a concession to the operating temperature of the sensor but the condition that makes an electrostatic measurement exist at all. The upper limit is then devitrification of the frozen sample near 130–150 K rather than any property of the sensor. The practical operating range of the device as designed is thus 0.3–19 K.

The magnetic field requirement differs between the two variants and is modest in both. The Coulomb-blockade dot in gapped bilayer graphene requires no field at all: confinement is electrostatic and the resonances are those of Coulomb blockade, so the sensitivity estimates of Section 4.1 are zero-field figures. The quantum-Hall point contact does require a field, but the √B dependence of the Landau spacing in graphene makes the requirement mild: ΔE = 51 meV at 2 T, 36 meV at 1 T and 26 meV at 0.5 T, the last exceeding kBT at 4.2 K by a factor of 71. The field at which this variant ceases to work is therefore set by disorder broadening rather than by temperature, and in h-BN-encapsulated graphene the quantum Hall regime is well developed below 1 T, where a compact permanent-magnet assembly or a small superconducting solenoid is sufficient and no large-bore magnet is required. The 2 T used in Figure 3 is an illustrative value well within the range of the solenoids fitted as standard to the cryostats described above, and not a lower bound on operation.

**Figure 7:**
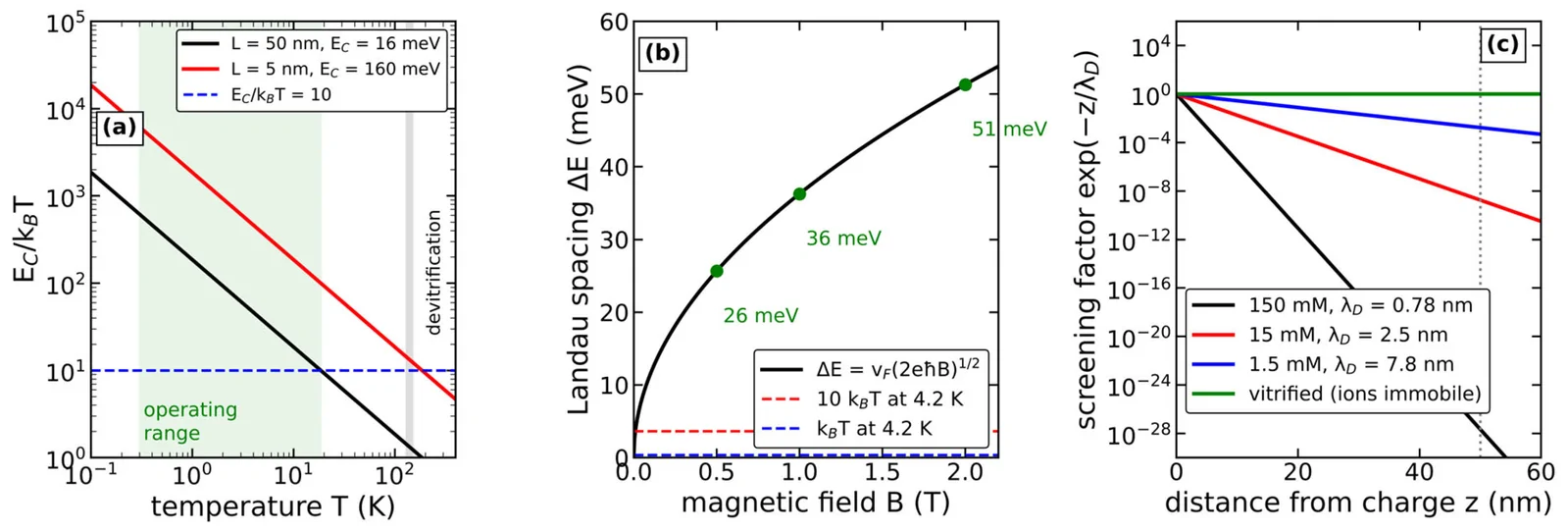
(**a**) Ratio E_C_/k_B_T as a function of temperature for the 50 nm island (E_C_ = 16 meV, black) and for a 5 nm island (E_C_ ≈ 160 meV, red). The blue dashed line E_C_/k_B_T = 10 sets the upper temperature of operation, about 19 K and 190 K respectively; the green band is the operating range 0.3–19 K of the present design and the grey band the devitrification range of the sample, 130–150 K. (**b**) Spacing ΔE between the N = 0 and N = 1 Landau levels of monolayer graphene, Equation (1), with the values at 0.5, 1 and 2 T, compared with k_B_T and 10k_B_T at 4.2 K. (**c**) Debye screening factor exp(−z/λ_D_) of the field of a charge in electrolyte at 300 K for ionic strengths of 150, 15 and 1.5 mM, compared with the vitrified sample, in which the ions are immobile (green). At z = 50 nm (dotted) and 150 mM the field is suppressed by about 28 orders of magnitude.

Figure 7 collects the three conditions that fix this operating window: the ratio E_C_/k_B_T, which sets the upper temperature of the sensor (a); the Landau spacing of monolayer graphene, which sets the field required by the quantum-Hall variant (b); and the Debye screening of the electrolyte, which makes the measurement possible only in the vitrified state (c).

### 5.5. Transitioning from Measured Offsets to Interfacial-Potential Maps

Reversing the measurement involves an ill-posed problem, since a set of offset charges Q_0,j_, one per element (Section 4), must be converted into a source distribution. The signal of the jth sensing element is related to the surface-proximal charge density σ(r) in the plane at height z_0_ by reciprocity, sj = ∫d^2^r σ(r) φw(j)(r, z_0_), where φw(j) is the weighting potential of that element—the same quantity that appears in Section 4.1. The forward kernel is therefore not postulated: it is the solution of a Laplace problem for the actual electrode geometry, including the split gates, the contacts, the h-BN stack and the back gate, and it is computed with the electrostatic solver of Section 5.1. For the electroneutral frozen sample of Section 4.2 the source is the dipole density p(r) and the kernel is ∂_z_φ_w_, Equation (5); in Fourier space this multiplies the charge kernel discussed below by q, so that the reconstructed quantity is the band-limited interfacial potential ψ_s_ + βV_m_. Its q = 0 component, which is in any case lost to the offset ambiguity noted below, carries no information. As pointed out in Section 4.1, the exponential attenuation also limits the depth range: the reconstruction must be seen as a two-dimensional representation of the surface-proximal charge density with a weak and rapidly decaying depth weighting, and not as a tomographic reconstruction of the interior of the cell. The reconstruction is achieved through Tikhonov regularisation of the discretised forward operator, with the regularisation parameter determined by the L-curve criterion [69,70]. It would be a natural long-term application to correlate the reconstructed charge maps with the membrane potential and disease associations listed in Ref. [7], but such a correlation would be hypothesis-generating rather than diagnostic and would first require the frozen-versus-living issue discussed above to be resolved.

It is possible to evaluate the conditioning of this inversion before any device is constructed. In Fourier space the forward operator is diagonal, and for orientation its transform factorises approximately as K(q) ≈ A(q) exp(−qz_0_)/εg(q), with A(q) the transfer function of the split-gate aperture, exp(−qz_0_) the geometric attenuation from the charge plane to the sheet, and εg(q) = 1 + qTF/q the static screening of the graphene itself. The aperture factor and the estimate exp(−πh/d) of Section 5.2 are the same quantity in two limits rather than independent approximations, since the field leaking through a slit of width d in a grounded plane decays away from that plane as exp(−πz/d). Because the weighting potential lies between zero and unity everywhere, by the maximum principle, K(q) is non-negative with |K(q)| ≤ K(0): the kernel is low-pass, and the ill-conditioning of the inversion lies entirely at large q, where exp(−qz_0_) and the finite lateral extent of each element suppress the response. At small q the limitations are not in the kernel but in the measurement: Fourier components with q < 2π/L are not sampled by an array of lateral extent L, and a spatially uniform offset cannot be distinguished from a drift of the gate working point and must be removed by common-mode rejection across elements. The reconstruction should accordingly be read as a band-limited, surface-weighted two-dimensional map whose absolute offset is unconstrained.

We have solved this Laplace problem numerically for the geometry of Section 5.2—graphene sheet and constriction at z = 0, split gates with an aperture of width d at height h, a grounded back gate, and relative permittivity 3 throughout—taking the constriction to be uniform along its length. The computed weighting potential is non-negative everywhere, as the maximum principle requires, and its transform is accordingly largest at q = 0. At z_0_ = 50 nm the peak weighting potential is 0.15, 0.38 and 0.64 for apertures of 50, 100 and 200 nm respectively, while the corresponding kernels fall to half power at periods of 220, 270 and 390 nm. The roll-off is thus considerably faster than that of the analytic form, and the resolution that follows is correspondingly coarser; the consequences are drawn out below. The solver uses a finite-difference discretisation with Dirichlet boundary conditions on the constriction, the split gates and the back gate; it was implemented with the assistance of Claude (Anthropic), and the authors verified the results independently, including the non-negativity of the weighting potential required by the maximum principle and the convergence of the computed coupling and kernel with grid spacing, which change by 2 and 3 per cent respectively between 2 nm and 1 nm grids. A three-dimensional calculation for an element of finite length is in progress and will reduce the quoted couplings.

**Figure 8:**
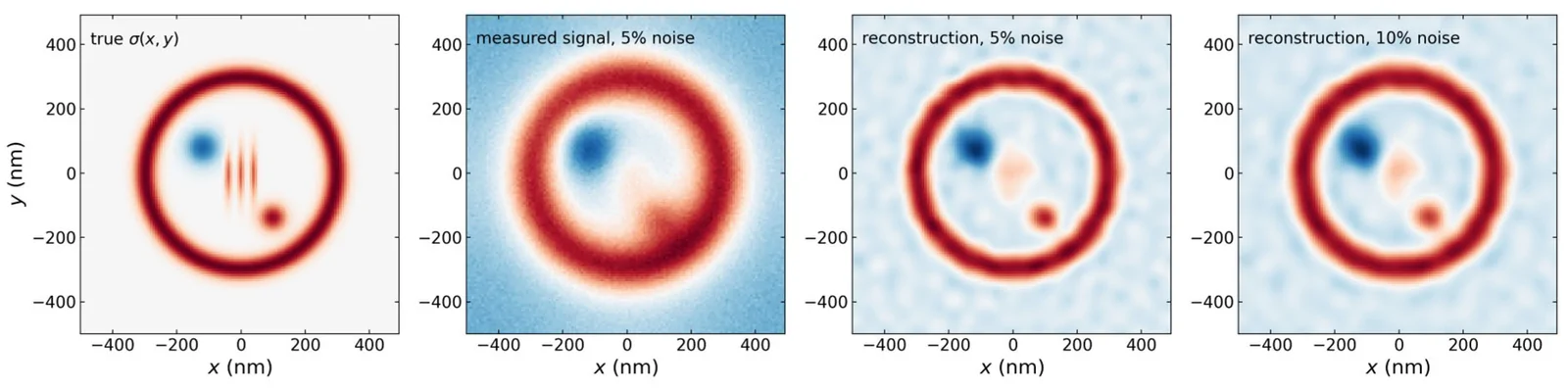
A synthetic forward–inverse test at z_0_ = 50 nm for dense sampling (kernel-limited bound). From left to right are shown the true charge map σ(x,y), the signal recorded at the sensor plane with 5% added noise, and the Tikhonov reconstructions for 5% and 10% noise levels. The 300 nm rim and the 60–80 nm patches are recovered with the correct position and sign; the 40 nm stripe triplet is not resolved at either noise level. With the 8 × 8 array at 250 nm pitch the 60–80 nm patches are not resolved either: they register only as signals in the elements beneath them (see text).

**Figure 9:**
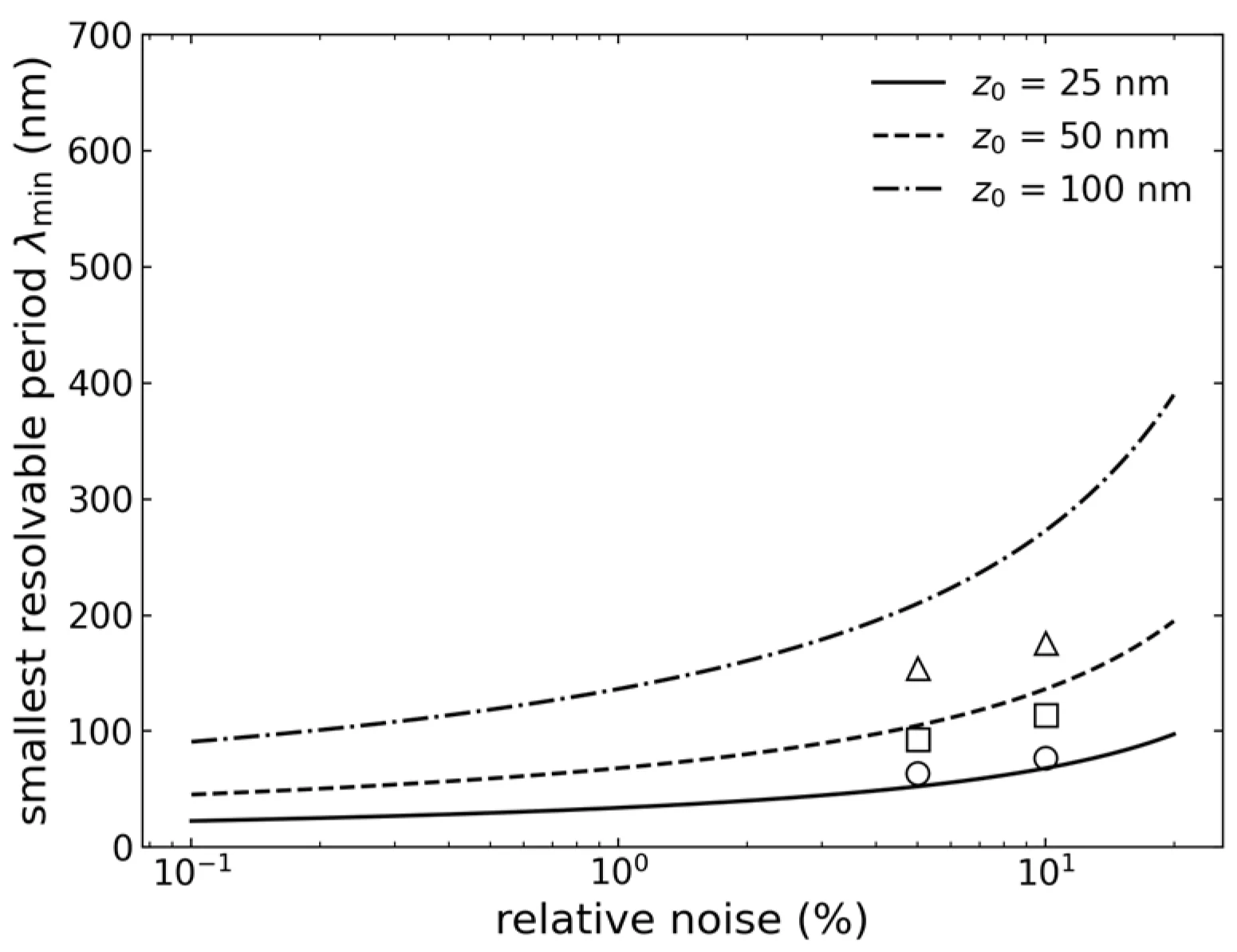
The smallest resolvable period as a function of the relative measurement noise. The lines show the analytic bound given by Equation (8) for three charge-to-sensor separations; the symbols represent the values obtained from the Tikhonov reconstructions in Table 1 (circles for z_0_ = 25 nm, squares for 50 nm, triangles for 100 nm).

For the resolution test we produced a synthetic charge map featuring a 300 nm membrane rim, two protein-scale patches of 60–80 nm and a triplet of 40 nm stripes, and inverted it by Tikhonov regularisation with the regularisation parameter fixed by the maximum curvature of the L-curve [69,70]. In order to avoid the inverse crime of generating and inverting with the same operator, the synthetic data are generated with the full numerical weighting potential of Section 5.1—including finite gate thickness, the multilayer dielectric stack and graphene screening—while the inversion uses the factorised analytic kernel above, so that model error is present by construction and can be separated from noise. Reported quantities are averaged over fifty noise realisations with seeds 1–50. The reconstruction grid is 512 × 512 over a field of view of 4 µm × 4 µm, giving 7.8 nm pixels; the field is sampled on the full reconstruction grid, so that the test gives the kernel-limited bound for dense sampling and does not include the 250 nm pitch of the 8 × 8 array, which is treated separately below; the boundary is treated periodically with a 25% zero-padded guard band to suppress wrap-around; the regularisation matrix is scaled to unit spectral norm before the L-curve is computed; and the L-curve corner is located by the maximum curvature of the spline-interpolated log–log residual–norm trade-off, the resulting parameter being reported for every case. Resolution is reported as the smallest resolvable period λ_min_ = 2π/q_c_, where q_c_ is the wavevector at which the effective Tikhonov filter falls to one half; this is the single definition used in Table 1 and Figure 8. Figure 9 displays the result obtained for z_0_ = 50 nm.

In this dense-sampling test the rim together with both patches have been recovered while retaining both their positions and signs, whereas the 40 nm stripes are missing at all the noise levels tested and instead appear as one smeared feature. Table 1 lists the smallest resolvable period λmin, which is defined as 2π/qc where qc is the wavevector at which the effective Tikhonov filter drops to one half, along with the relative L2 error of the reconstruction.

The cutoff that has been recovered is based on a simple bound; if you set the attenuation exp(−qz_0_) equal to the relative noise level ε, then you obtain the largest wavevector which carries information above the noise floor, and hence

(8) λmin = 2πz_0_/ln(1/ε),

Equation (8) is a noise-floor bound and reproduces the numerical cut-off only in the middle of the range studied: the ratio of the numerical λmin to the bound falls from 1.2 at z_0_ = 25 nm through 0.9 at 50 nm to 0.6 at 100 nm. At small separations the reconstruction grid rather than the noise sets the finest recoverable period, while at large separations the bound is conservative relative to a detection-based criterion. Equation (8) should therefore be read as an order-of-magnitude scaling—λmin linear in z_0_ and only logarithmic in the signal-to-noise ratio—and not as a quantitative prediction. The design conclusion drawn from it is unaffected, since it rests on the two scalings rather than on the prefactor. Figure 9 presents a comparison of the two.

Two consequences follow, and they set the design priorities. The first is a scaling: the resolution is linear in the separation z_0_ but only logarithmic in the signal-to-noise ratio, so that improving the noise from 10 per cent to 1 per cent gains a factor of about 1.4 in λmin, whereas halving the h-BN thickness gains a factor of two. Effort invested in thinning the dielectric stack is therefore more worthwhile than effort spent on the readout chain.

The second is a correction to the resolution anticipated in Section 4, and it is the more important of the two. The values in Table 1 were obtained with the analytic kernel in the inversion step and with dense sampling, and they take no account of the pitch at which the array samples the plane; both omissions matter. The numerically computed kernel of the actual split-gate geometry falls to half power at a period of about 270 nm at z_0_ = 50 nm for an aperture d = 100 nm, rather than at the 94 nm of Table 1, and since the element pitch cannot be smaller than the aperture, the sampling theorem imposes a second limit of twice the pitch, which is of the same order. The achievable period is therefore of order 250 to 300 nm at z_0_ = 50 nm, and not 100 nm. The aperture enters the design twice and in opposite directions: a narrower aperture samples the plane more finely and gives a sharper kernel, but couples more weakly to the charge, the computed weighting potential at z_0_ = 50 nm falling from 0.64 for d = 200 nm through 0.38 for d = 100 nm to 0.15 for d = 50 nm. This trade-off, rather than the exponential attenuation alone, is the central constraint on the design, and the values in Table 1 should be read as the kernel-limited bound rather than as an achievable resolution.

One limitation of this test should be mentioned. The noise varied here is instrumental noise on the measured transconductance, whereas in practice the dominant source of error will be model error—an incorrect effective permittivity, gate geometry or separation in the assumed kernel. The protocol above addresses this by using different operators in the forward and inverse steps, but the residual model error has not been varied systematically, and the figures in Table 1 should therefore be regarded as an upper limit on the performance that can be achieved. Table 1 also carries internal evidence that the reported errors are not noise-limited: at z_0_ = 25 nm the relative L2 error is 0.43 at both 5% and 10% noise, which indicates that at small separations the error is set by discretisation and by the bias of the regularisation rather than by the noise. The electrical noise of the device itself—shot noise and flicker noise in the graphene—is treated separately in Section 4.1.

## 6. Conclusions

We have analysed the design of a non-contact sensor array that images, with sub-micrometre resolution, the interfacial charge layers of a single vitrified cell. The key idea is that a Coulomb-blockade quantum dot in gapped bilayer graphene exhibits a sharp and tunable response to the electric stray field of a nearby cell; the monolayer quantum-Hall point contact is an alternative realisation of the same principle, to which no figure of merit is attached here. The benefit is not in the absolute capacitance—since the total capacitance, which is the series combination of geometric and quantum capacitance, is limited above by the geometric capacitance, it is impossible to construct a small probe that achieves a large capacitance through quantum effects—rather, it lies in the slope: near a sharp resonance in the density of states—a Landau level in the monolayer point contact, a Coulomb-blockade level in the gapped-bilayer dot—the derivative of the capacitance with respect to the Fermi energy is large, and it is this derivative that determines the transconductance and thus the sensitivity. Since this derivative is a characteristic of the density of states and not of the device area, and since the lever arm that couples the cell to the device is approximately scale-invariant, sensitivity and probe size are independent of each other. The estimate given in Section 4.1, which refers to the quantum-dot variant, shows that the response is about one resonance linewidth per elementary charge at a separation of 50 nm and at 4.2 K; the amplifier-limited charge noise is 2.5 × 10^−3^ e/√Hz, degrading to an assumed 1/f-limited 10^−2^ e/√Hz in the band of interest, comparable to that obtained by scanning single-electron-transistor electrometry [20] but at a smaller sensor-to-charge distance and without the need for a scanned probe. The inversion process by which the measured currents are converted into a charge map is quantified in Section 5.5: at a separation of 50 nm the array resolves a period of order 250 to 300 nm, set jointly by the roll-off of the numerically computed kernel of the split-gate geometry and by the pitch at which the array samples the plane. This degrades linearly with increasing separation but only logarithmically with increasing noise, so that the resolution of the method is determined by the geometry of the stack and of the array rather than by the noise level of the readout. Finally, the measured quantity has been identified: because the frozen sample is electroneutral, the array images lateral variations in the interfacial dipole density, that is of ψ_s_ + βV_m_ with β ≈ 0.07, Equation (4). A 10 mV surface-potential contrast at 250 nm period is detected with a margin of about one order of magnitude in 1 ms, and the membrane potential is accessible only at the cell edge and through the calibration series of Section 5.3.

Understanding the concept demands advancement in a number of areas. In order to enable a measured current to be converted into a charge distribution, the self-consistent quantum-electrostatic framework which has been developed for semiconductor heterostructures must be extended to accommodate graphene’s Dirac spectrum. Graphene quantum point contact (QPC) and quantum dot (QD) devices, defined by electron-beam lithography and enclosed in h-BN, offer a suitable platform on which the cells can be placed directly, thus reducing the separation that affects both resolution and signal; since the stray field decreases according to exp(−qz), this close-proximity arrangement is not merely incidental but is in fact essential, and it is also the reason for the split-gate geometry mentioned in Section 5.2. Cryopreservation protocols exist that vitrify thin cell layers without lethal ice formation, but whether they preserve the charge distribution at the sensor interface—as distinct from preserving viability, which is what those protocols were developed to optimise—has not been established, and is a prerequisite for this measurement rather than a consequence of it.

The following questions are still open and are presented as such. In the case of monolayer graphene, confinement is due to the magnetic field and not to the gates, so the QPC version is only well defined in the quantum Hall regime; a Coulomb-blockade quantum dot on the other hand requires a gap and therefore has to use bilayer graphene [32], since the smaller Landau spacing in bilayer graphene in part cancels out the advantage that was the original reason for using graphene. There is still no solution to accurately and repeatedly position the cell with respect to the sensor, as the cell cannot be moved after it has been frozen; lateral coverage must be achieved by an array of constrictions on a single sheet. The sensitivity estimate given in Section 4.1 is based on an approximate treatment of the dielectric screening of the frozen cell and of the split-gate aperture, and calls for direct experimental verification; no transport data are included in this text. The relationship between the frozen charge distribution and the electrophysiology of the living cell, as discussed in Section 5.3, is the point on which any biological interpretation must ultimately rest. Room-temperature operation, for which the large Landau-level spacing of graphene is the main justification, remains the most difficult objective, and since quantum Hall physics has so far only been observed at fields of the order of 30 T [23], it is a long-term rather than a near-term possibility.

Even though there are still some open questions, this method does provide a physically well-justified path to sub-micrometre, non-contact imaging of the interfacial charge layers of cells. Since it bases sensitivity on a quantum mechanical rather than a geometric feature of the sensor, it would achieve a combination of spatial resolution and non-contact operation which classical capacitive and impedance-based techniques are unable to attain at the same time.

## Figures and Tables

**Table 1:** The smallest resolvable period λmin and the relative L2 error of the reconstructed charge map for three values of the charge-to-sensor separation z_0_ and two levels of measurement noise, as obtained from the synthetic test described in the text.

z_0_ (nm)	Noise (%)	λmin (nm)	Relative L2 Error
25	5	64	0.43
25	10	77	0.43
50	5	94	0.44
50	10	114	0.46
100	5	155	0.54
100	10	176	0.58

## Data Availability

The study contained no new experimental data.
